# Curriculum Development: A National Curriculum for Physician Associates in Mental Health

**DOI:** 10.1192/bjo.2022.138

**Published:** 2022-06-20

**Authors:** Pranav Mahajan, Helen Crimlisk, Ellie Wildbore, Paris Tatt-Smith, Tony Roth

**Affiliations:** 1 Health Education Yorkshire & Humber, Sheffield, United Kingdom.; 2 Royal College of Psychiatrists, London, United Kingdom; 3 Sheffield Health and Social Care NHS Foundation Trust, Sheffield, United Kingdom; 4 Royal College of Psychiatrists, London, United Kingdom.; 5 National Collaborating Centre for Mental Health, London, United Kingdom

## Abstract

**Aims:**

Physician associates (PAs) are becoming more commonplace in psychiatric services in the UK to help address long term workforce difficulties. The 2019 NHS Long Term Plan detailed a commitment to transforming mental health care in England recognising that services were not meeting current or future increase in demand. Health Education England's (HEE) report, Stepping Forward to 2020/21: The Mental Health Workforce Plan for England, described a longer-term strategy to expand the mental health workforce, including recruiting 5,000 people into ‘new roles’ including physician associates. The NHS Mental Health Implementation Plan 2019/20–2023/243 stated an aim of recruiting 140 PAs to the workforce over five years in addition to the requirements specified in the HEE report. A curriculum for PAs working in mental health would set out the competencies required to work in mental health services.

**Methods:**

The curriculum was developed by the National Collaborating Centre for Mental Health (NCCMH). The work was overseen by an expert reference group, comprising experts in training PAs in mental health, PAs, researchers and experts by experience, all selected for their expertise in research, training and service delivery.

**Results:**

The overarching aims and objectives of the curriculum was to convey a practical understanding of the attitudes, knowledge and skills that underpin the role, thus enabling PAs to offer effective and value-driven support to patients.

The completed curriculum has been arranged into seven modules: Knowledge, Professional/Legal Issues, Engagement and Communication, Diagnostic Assessment and Treatment Planning, Interventions, Managing the Interface of Mental and Physical Health and Team Working. This reflects the expected roles and responsibilities of PAs working in mental health.

**Conclusion:**

HEE and the Royal College of Psychiatrist have recommended all mental health organisations implement an educational programme for new PAs. The curriculum will inform the training requirements for PAs and standardise the training they receive from mental health organisations. It should support the work of PA supervisors and peer coordinators, and those delivering education and training to them. The curriculum will be a dynamic document and work will be needed to adapt it as the role changes, for example with incoming regulation and potential prescribing rights that follow.

